# Contribution of Diabetes to the Incidence and Prevalence of Comorbid Conditions (Cancer, Periodontal Disease, Fracture, Impaired Cognitive Function, and Depression): A Systematic Review of Epidemiological Studies in Japanese Populations

**DOI:** 10.2188/jea.JE20170155

**Published:** 2019-01-05

**Authors:** Hirokazu Tanaka, Noriko Ihana-Sugiyama, Takehiro Sugiyama, Mitsuru Ohsugi

**Affiliations:** 1 Diabetes and Metabolism Information Center, Research Institute, National Center for Global Health and Medicine, Tokyo, Japan; 2 Department of Public Health, Graduate School of Medicine, the University of Tokyo, Tokyo, Japan; 3 Department of Diabetes, Endocrinology and Metabolism, National Center for Global Health and Medicine Center Hospital, Tokyo, Japan

**Keywords:** diabetes mellitus, diabetes complications, epidemiological studies, review, Japan

## Abstract

**Background:**

Several epidemiological studies have determined the relationship between diabetes and the incidence and/or prevalence of recently identified comorbid conditions (cancer, periodontal disease, fracture, cognitive impairment, and depression). These relationships may vary by country or race/ethnicity. We aimed to systematically review studies in this field conducted with the Japanese population because such a review in the Japanese population has never been undertaken.

**Methods:**

We conducted systematic literature searches in PubMed and Ichushi-Web databases for studies published until December 2016. Studies comparing the incidence and/or prevalence of the comorbidities among the Japanese population were included. The studies were classified as integrated analyses, cohort studies, case-control studies, or cross-sectional studies.

**Results:**

We identified 33 studies (cancer: 17, periodontal disease: 5, fracture: 5, cognitive impairment: 4, and depression: 2). Although several cohort studies and meta-analyses had assessed the development of cancer in diabetes, there was scant epidemiological evidence for the other conditions. Indeed, only one cohort study each had been conducted for periodontal disease, fracture, and cognitive impairment, whereas other evidence was cross-sectional, some of which was induced from baseline characteristic tables of studies designed for other purposes.

**Conclusion:**

In Japan, there is insufficient evidence about the relationship between diabetes and the incidence/prevalence of periodontal disease, fracture, cognitive impairment, and depression. By contrast, several cohort studies and integrated analyses have been conducted for the relationship with cancer. Further studies should be undertaken to estimate the contribution of diabetes on the incidence/prevalence of comorbidities that may be specific to the Japanese population.

## INTRODUCTION

Diabetes mellitus has become increasingly common, and it is known that inadequate disease control can undermine quality of life through diabetes-specific symptoms^1^ and conventional diabetic microvascular complications.^2^ In addition, macrovascular complications, such as coronary heart disease and stroke, occur more frequently in patients with diabetes and are the leading causes of death for those with diabetes in many countries.^3^ With their increased prevalence, diabetes and its associated complications are now regarded as a global burden.^3^ Thus, reducing the prevalence and economic burden of diabetes has become an important goal of medical care and health policy.^4^^,^^5^

Recently, comorbidities, such as cancers, fractures, and dementia, have been viewed as diabetes-related and can impair both the quality of life and the survival of patients.^2^ Given that the life expectancy of patients with diabetes has been prolonged because of improved care for conventional major diabetic complications,^6^ these newly recognized comorbidities and the increase in the number of elderly patients with diabetes have emerged as new healthcare challenges. Comparing the incidence and prevalence of these conditions between individuals with diabetes and those without is required to assess whether diabetes prevention or its management helps prevent these conditions.

Some studies have attempted to reveal the relationship between diabetes and these emerging comorbidities in the Japanese population. However, we suspect that these newly identified comorbidities are poorly captured in epidemiological studies and that their burden is large in Japan; the number of patients with diabetes was estimated at 7.2 million adults in 2015.^4^ In addition, we considered that the most of evidence that was shown in the Japanese guideline for diabetes care was produced in other countries.^7^ Crucially, the relationship between diabetes and these newly identified diabetes-related conditions may vary by country or ethnicity. Within this context, in this systematic review, we aimed to summarize the current research about the incidence and prevalence of the newly recognized diabetes-related conditions among patients in Japan.

### Scope

Among the conditions that appear related to diabetes, we focused on five: cancer, periodontal disease, fracture, impaired cognitive function/dementia, and depression. These were selected for the following reasons: (1) the pathophysiological relationships between diabetes and the complications are well established; (2) epidemiological evidence has been reported linking diabetes and those conditions, at least outside of Japan; (3) the burdens of those comorbidities on quality of life, complexity of medical care, and/or longevity of patients are significant; (4) preventing or treating diabetes has been shown to improve outcomes in those complications, or there are remedies for treatment or alleviation of symptoms; and (5) the potential impact of revealing the incidence and prevalence of those conditions is considerable in the areas of epidemiology, medical economics, and diabetes management. The American Diabetes Association guideline referred to six other comorbidities (autoimmune diseases, fatty liver disease, hearing impairment, human immunodeficiency virus, low testosterone in men, and obstructive sleep apnea). However, we excluded these comorbid conditions because they did not meet the criteria above. For instance, we excluded fatty liver disease due to the following reasons: it results from multiple factors, including patients’ lifestyle, and not solely from diabetes; it does not directly influence the quality of life; and it does not require specific medical care, unless it develops into cirrhosis or liver cancer. Although the scope of our research was limited to the Japanese population, we first summarize the pathophysiological basis for including the selected complications, together with the epidemiological data.

### Cancer

There is a growing body of evidence that diabetes is associated with an increased incidence of cancer, which is supported by a consensus report from the American Diabetes Association and the American Cancer Society in 2010.^8^ Many mechanisms have been proposed to explain the link between increased cancer risk and diabetes, including insulin resistance and hyperinsulinemia, increased oxidative stress caused by hyperglycemia, chronic inflammation, and abnormalities in the levels of adipocytokines.^9^ However, epidemiological evidence is not straightforward to interpret because risk factors (eg, age, sex, obesity, and inappropriate diets) may confound the relationship.^9^ An umbrella review of 27 meta-analyses looking at the relationship between type 2 diabetes and cancer incidence or mortality was published in 2015.^10^ All of the original 27 meta-analyses reported increased risks of developing cancer among those with diabetes; however, the review found that less of the cancer incidence could be significantly associated with type 2 diabetes after considering the substantial heterogeneity, small study effects, and excess significance in the studies.^10^ We focused on the development of cancer in patients with diabetes in Japan.

### Periodontal disease

Periodontitis refers to chronic inflammation of the gingiva, which leads to periodontal pocket formation, loss of connective tissue attachment, and alveolar bone resorption. There are several good-quality studies showing the relationship between diabetes and periodontal disease; for example, in a large-scale cohort study of Pima Indians in the United States, the rate of periodontal disease in those with type 2 diabetes was 2.6 times higher than in those who had no periodontal disease.^11^ There was another useful analysis by the National Health and Nutrition Examination Survey III in the United States. This cross-sectional population-representative survey showed that, compared to patients without diabetes, those with poorly controlled (HbA1c >9.0%) and moderately controlled (HbA1c ≤9.0%) type 2 diabetes had 2.9-fold and 1.6-fold higher rates of periodontitis, respectively.^12^ The causal relationship between diabetes and periodontitis is considered bidirectional, with periodontitis being associated with whole-body insulin resistance^13^ and diabetes and poor glycemic control being known risk factors for development and progression of periodontitis.^14^ We focused on the causal direction from diabetes to periodontal disease in Japanese patients.

### Fracture

Diabetes affects bone metabolism and structure, making it a potential risk factor for fracture and osteoporosis. It has been recognized that oxidative stress and advanced glycation end products caused by hyperglycemia play important roles in deteriorating bone quality.^15^ According to meta-analyses of studies from Western and East Asian countries, the association between diabetes and hip fracture was stronger among patients with type 1 diabetes (relative risk [RR], 6.3–6.9) than among those with type 2 diabetes (RR, 1.4–1.7).^16^^,^^17^ A discrepancy in the effect of diabetes on bone mineral density (BMD) between type 1 and type 2 diabetes has also been reported, with evidence that spine and hip BMD decreased among patients with type 1 diabetes but increased among those with type 2 diabetes.^16^ Regardless of BMD, increased fragility of bone structure increases the risk of fracture in patients with diabetes. We focused on the incidence and prevalence of fracture among patients with diabetes in Japan.

### Impaired cognitive function and dementia

The relationship between diabetes and major types of dementia has been discussed in epidemiologic studies and pathophysiological studies.^18^ Diabetes and its associated cardiovascular risk factors result in an increased risk of atherosclerosis and stroke, both of which can cause vessel degeneration in the brain. Hyperglycemia-induced glucose toxicity causes microvascular abnormalities and brain aging. Furthermore, diabetes and its treatment may induce abnormal amyloid metabolism, which can cause Alzheimer-type pathology.^18^ In a recent study in mainly non-Japanese subjects, patients with type 2 diabetes were shown to be at a significantly higher risk of dementia. In that study, the multiple-adjusted pooled RR for any form of dementia being associated with diabetes was 1.73 in men and 1.62 in women.^19^ In this study, we review the available literature on the incidence and prevalence of impaired cognitive function and dementia among patients with diabetes in Japan.

### Depression

Depression is common in patients with diabetes. An international meta-analysis has shown that the odds of having depression among patients with diabetes are double those of people without diabetes.^20^ The relationship between diabetes and depression is thought to be bidirectional: depression is a risk factor for developing diabetes, but diabetes is also a risk factor for developing and exacerbating depression. A possible pathophysiological explanation for the causal link between depression and diabetes is dysregulation of key regulatory systems, including the hypothalamic-pituitary-adrenal axis and the sympathoadrenal system, and the increased production of proinflammatory cytokines associated with insulin resistance.^21^ By contrast, diabetes and its complications are also implicated as risk factors for depression,^22^ presumably because of the stress caused by the diagnosis or complications of diabetes and the side effects of antidiabetic drugs.^23^ Based on pooled data of 48,808 patients with diabetes, the presence of type 2 diabetes was associated with a 24% increased risk of having depression^24^; however, the mechanisms underlying this remain unclear. In this literature review, we focused on diabetes as a risk factor for depression, excluding studies investigating the causal link from depression to developing or aggravating diabetes.

## MATERIAL AND METHODS

### Search strategy

We followed the PRISMA Statement (please see eMaterial 1)^25^; this study has not been registered on the PROSPERO database. We conducted a literature search of the MEDLINE (PubMed) and Ichushi-Web databases (a Japanese database for literature search) for studies published until December 31, 2016. PubMed searches were also conducted using medical subject headings terms. For a start, we searched the literature by including all of the following four words or phrases: “diabetes,” “prevalence or incidence,” “Japan,” and the name of the comorbid condition of interest (eg, “cancer”). If the number of identified studies was fewer than 50 using this method, we repeated the search excluding “prevalence” or “incidence” from the list of search terms. Regarding fracture, we found that including the term “fracture” did not capture the required studies well, and these studies were detected by adding the word “osteoporosis” instead; therefore, we added the term “osteoporosis” to increase coverage. Other search terms used in PubMed are detailed in eTable 1.

For the systematic review, literature was selected in three steps. First, we screened article titles. Second, we checked the content of the abstracts to judge their relevance. Third, we selected articles by reading the whole text of suitable articles. At each step, two authors (HT and NIS) independently extracted the literature, and any disagreement regarding selection was resolved via consensus among all authors. Additional articles were identified using citation tracing in the included articles.

### Study selection and evidence classification

In the current review, we focused on epidemiological studies comparing the incidence and prevalence of the conditions of interest in the general population of Japan. We excluded studies not conducted in Japanese populations and review articles that did not include a meta-analysis. Main measures were set as hazard ratio (HR), incidence rate ratio (IRR), and odds ratios (ORs). The term “relative risk (RR)” used in each reviewed study was carefully converted to one of the following: HR, IRR, and OR, in order to avoid ambiguity. Studies that used disease-specific mortality to confirm disease were excluded because the estimated RR would be influenced by the different incidence rates for developing the comorbid disease and by the different mortality rates among patients developing that disease. We focused solely on the causal direction from diabetes to common comorbidities, so we excluded longitudinal studies that intended to reveal the effect of those comorbidities on developing diabetes.

For the cross-sectional analyses, we included not only typical cross-sectional studies that investigated the relationship between diabetes and the target disease but also the baseline characteristics in tables of study participants in other studies, regardless of the research questions but provided the study setting did not deviate too far from the general population and provided the cross-sectional relationship between diabetes and the disease could be investigated using prevalence ORs.

After screening the identified literature, we classified epidemiologic studies into the following categories: meta-analyses/systematic reviews (ie, integrated analyses), cohort studies, case-control studies (longitudinal), and cross-sectional studies (Table 1). We excluded single-arm cohort studies that included only patients with diabetes and retrospective cohort studies that examined the prevalence of diabetes in only “cases” (ie, those who suffered from comorbid conditions). This was because comparison between the groups within a study would probably have ensured some degree of comparability. Moreover, regardless of whether a study was called a case-control study by its authors, we classified it as a cross-sectional study if the exposure and outcome were measured simultaneously. Regarding cohort and case-control studies, we evaluated the quality using the Newcastle-Ottawa scale (eTable 2 and eTable 3).^26^ In this study, we did not conduct a meta-analysis because we found few epidemiological studies regarding diabetes-related conditions in the Japanese population.

**Table 1.** Evidence classification

**I**	**Integrated analyses**	Any meta-analyses of cohort and case-control studies

**II**	**Cohort study design**	Comparison of the incidence of a given comorbid condition between patients with and without diabetes. The incidence rate ratio of the condition is estimated or can be estimated regarding diabetes exposure. There is no distinction between prospective and retrospective studies.

**III**	**Case-control study design (longitudinal)**	Comparison of the proportion with a history of diabetes among those who did and did not experience a given comorbid condition. The odds ratio of the condition is estimated regarding diabetes exposure. The timing of exposure (diabetes) and the outcome measurement should be separate (longitudinal).

**IV**	**Cross-sectional study design**	Comparison of the prevalence of a given comorbid condition between patients with and without diabetes. The prevalence ratio or odds ratio is either estimated or can be estimated.

## RESULTS

### Cancer and diabetes

We reviewed 67 titles and abstracts of studies looking at the relationship between cancer and diabetes. Among them, we identified three integrated analyses,^27^^–^^29^ eight cohort studies,^30^^–^^37^ five case-control studies,^38^^–^^42^ and one cross-sectional study (Table 2).^43^

**Table 2.** Number of studies identified through literature search

Evidence classification^a^	I	II	III	IV
Cancer	3	8	5	1
Periodontal disease	0	1	0	4
Fracture	0	1	0	4
Impaired cognitive function	0	1	0	3
Depression	0	0	1	1

^a^I: Integrated analyses, II: Cohort study design, III: Case-control study design (longitudinal), IV: Cross-sectional study design.

A pooled analysis of eight cohort studies showed that diabetes was associated with statistically significant increased risks of all cancers (HR 1.19; 95% confidence interval [CI], 1.12–1.25), colon cancer (HR 1.40; 95% CI, 1.19–1.64), liver cancer (HR 1.97; 95% CI, 1.65–2.36), pancreatic cancer (HR 1.85; 95% CI, 1.46–2.34), and bile duct cancer (HR 1.66; 95% CI, 1.14–2.41; men only).^27^ Another meta-analysis showed that the risk for all cancers was increased in men and women (OR 1.70; 95% CI, 1.38–2.10), though some of the studies only ascertained the incidence of cancer from death certificates. However, in a sex-specific analysis, diabetes was associated with a significantly increased risk of all cancers in men (adjusted RR 1.25; 95% CI, 1.06–1.46) and a borderline risk increase in women (adjusted RR 1.23; 95% CI, 0.97–1.56) based on three cohort studies and one case-control study.^28^ As for site-specific cancer risk, a systematic review was reported of liver cancer in diabetes, and it showed that the IRRs of diabetes for liver cancer were 2.10 (95% CI, 1.60–2.76) in cohort studies and 2.32 (95% CI, 1.73–3.12) in case-control studies.^29^

Among eight cohort studies, four analyzed all cancer risk and four analyzed site-specific cancer risk.^30^^–^^33^ A representative example of a study examining cancer risk is the Japan Public Health Center-Based Prospective study (JPHC study). In that study, 97,771 community-dwelling Japanese people aged 40–69 years were followed up for 13 years, and there was a 27% increased risk of total cancer observed in men among those with a history of diabetes compared with those without (*n* = 3,907 [366 with diabetes]; HR 1.27; 95% CI, 1.14–1.42).^30^ Specific cohort studies have reported site-specific cancer risks, including increased risks of pancreatic,^34^ gastric,^35^^,^^36^ and prostate^37^ cancer, in patients with diabetes. Case-control studies have also reported increased ORs of all,^38^ hepatocellular,^39^^,^^40^ pancreatic,^41^ and ovarian cancer.^42^ In a study reported as being case-control, but in which the exposure and outcome were measured simultaneously (so it was treated as a cross-sectional analysis in this study), there was an elevated OR for uterine endometrial cancer among patients with diabetes (Table 3).^43^

**Table 3.** Evidence table^a^

Reference (Publication year)	Design	Study population	Exposure measurement (diabetes)	Outcome measurement	Main results within the scope of the current literature review	Evidence classification^c^	Remarks
**Cancer**							
Sasazuki et al^27^ (2013)	Pooled analysis of 8 Japanese cohort studies	156,917 men and 182,542 women	Self-reported	The incidence of cancer (total and site-specific)	All-site cancer: HR: 1.19 (95% CI: 1.12–1.25)	I	Research Group for the Development and Evaluation of Cancer Prevention Strategies in Japan
Noto et al^28^ (2010)	Meta-analysis	4 cohort studies and 1 case-control study (250,479 subjects)	Type 2 diabetes	Cancer incidence in patients with or without diabetes	All-cancer risk: OR: 1.70 (95% CI: 1.38–2.10), adjusted RR: 1.25 (95% CI: 1.06–1.46) for men and 1.23 (95% CI: 0.93–1.56) for women	I	
Tanaka et al^29^ (2014)	Systematic review	19 cohort studies, 1 pooled analysis of 7 cohort studies, and 7 case-control studies	Diabetes	Liver cancer	IRR: 2.10 (95% CI: 1.60–2.76) from cohort studies, 2.32 (95% CI: 1.73–3.12) from case-control studies	I	
Inoue et al^30^ (2006)	Cohort study for 13 years (average follow-up: 10.7 years)	46,548 men (3,097 exposed and 43,451 unexposed) and 51,223 women (1,571 exposed and 49,652 unexposed)	Self-reported	Population-based cancer registry	Adjusted HR: 1.27 (95% CI: 1.14–1.42, all-site)	II	The JPHC study
Goto et al^31^ (2016)	Cohort study for 10 years (median follow-up: 8.5 years)	1,037 exposed (HbA1c ≧ 6.5%) and 8,314 unexposed (HbA1c: 5.0–5.5%)	HbA1c < 5.0%, 5.0–5.4%, 5.5–5.9%, 6.0–6.4%, ≧6.5%	Population-based cancer registry	Incidence rate: 13.7 for men, 5.7 for women per 1,000 people	II	
Nakamura et al^32^ (2013)	Cohort study for 16 years (average follow-up: 13.2 years)	14,173 men and 16,547 women	Self-reported	Population-based cancer registry	Adjusted HR for all-site cancer: 1.09 (95% CI: 0.93–1.29)	II	The Takayama study
Khan et al^33^ (2006)	Cohort study for 9 years (average follow-up: 8.1 years for men and 8.0 years for women)	23,378 men (1,753 exposed and 21,625 unexposed) and 33,503 women (1,554 exposed and 31,949 unexposed)	Self-reported	Population-based and hospital-based cancer registry	Adjusted IRR for liver cancer: 2.30 (95% CI: 1.47–3.59) for men and 2.70 (95% CI: 1.20–6.05) for women	II	The JACC study
Luo et al^34^ (2007)	Cohort study for 13 years (average follow-up: 10.7 years)	47,499 men and 52,171 women	Self-reported	Population-based cancer registry (pancreatic cancer)	Adjusted HR for pancreatic cancer: 2.1 (95% CI: 1.3–3.5) for men and 1.5 (95% CI: 0.7–3.5) for women	II	The JPHC study
Ikeda et al^35^ (2009)	Cohort study for 14 years	101 exposed (HbA1c ≧ 7.0%) and 1,685 unexposed (HbA1c: 5.0–5.9%)	HbA1c level	Medical history of gastric cancer	Adjusted HR (reference: HbA1c: 5.0–5.9%): 2.69 (95% CI: 1.24–5.85) for HbA1c ≧ 7.0%	II	The Hisayama study
Sekikawa et al^36^ (2014)	Historical cohort analysis for 9 years	148 exposed and 1,301 unexposed	Medical history	Endoscopic examination	Adjusted HR for gastric cancer: 2.76 (95% CI: 1.17–6.49)	II	
Li et al^37^ (2010)	Cohort study for 9 years (average follow-up: 7.8 years)	1,645 exposed and 20.813 unexposed	Self-reported	Miyagi Prefecture Cancer Registry	Adjusted HR: 1.18 (95% CI: 0.76–1.83) for all prostate cancer cases and 1.89 (95% CI: 1.02–3.50) for advanced cases	II	The Ohsaki Cohort Study
Kuriki et al^38^ (2007)	Hospital-based case-control study	19,540 men (5,341 cases and 14,199 controls) and 39,990 women (6,331 cases and 33,569 controls)	Self-reported	Hospital record and population-based cancer registry	Adjusted OR for all-site cancer: 1.44 (95% CI: 1.28–1.62) for men and 1.39 (95% CI: 1.19–1.62) for women	III	The Hospital-based Epidemiologic Research Program at Aichi Cancer Center
Ohishi et al^39^ (2008)	Nested case-control study	224 cases and 644 controls	Self-reported	The Hiroshima Tumor and Tissue Registry, Nagasaki Cancer Registry (hepatocellular carcinoma)	Adjusted OR for hepatocellular carcinoma: 1.98 (95% CI: 0.63–6.27)	III	The Adult Health Study longitudinal cohort (cohort of atomic bomb survivors)
Matsuo et al^40^ (2003)	Hospital- and community-based case-control study	354 men (177 cases and 177 community controls) and 90 women (45 cases and 45 community controls)	Hospital records	Medical record of Kurume University Hospital	Adjusted OR for hepatocellular carcinoma: 2.52 (95% CI: 1.27–5.02) for men and 4.20 (95% CI: 0.81–21.8) for women	III	
Inoue et al^41^ (2003)	Hospital-based case-control study	1,342 men (122 cases and 1,220 controls) and 858 women (78 cases and 780 controls)	Self-reported	Hospital record and population-based cancer registry (pancreatic cancer)	Adjusted OR for pancreatic cancer: 2.07 (95% CI: 1.14–3.74) for men and 1.29 (95% CI: 0.46–3.56) for women	III	The Hospital-based Epidemiologic Research Program at Aichi Cancer Center
Mori et al^42^ (1998)	Hospital- and community-based case-control study	89 cases and 323 controls	Self-reported	Hospital record	Adjusted OR for ovarian cancer: 3.21 (95% CI: 1.11–9.30)	III	
Inoue et al^43^ (1994)	Case-control study, but cross-sectional study design	286 women (143 cases and 143 controls)	Medical record	Clinically diagnosed (uterine endometrial cancer)	Adjusted OR for uterine endometrial cancer: 7.75 (95% CI: 1.52–40.0)	IV	

Reference (Publication year)	Design	Study population	Exposure measurement (diabetes)	Outcome measurement	Main results within the scope of the current literature review	Evidence classification^c^	Remarks

**Periodontal disease**							
Morita I et al^44^ (2012)	Cohort study for 5 years	5,856 participants	HbA1c ≥ 6.5%	Community Periodontal Index, 3 or 4	Adjusted RR: 1.17 (95% CI: 1.01–1.36)	II	
Marugame T et al^45^ (2003)	Hospital-based case-control study but cross-sectional analysis	664 men	Fasting plasma glucose ≥ 7.0 mmol/L and/or 2-h plasma glucose ≥ 11.1 mmol/L	Alveolar bone loss	Adjusted OR: 2.55 (95% CI: 0.86–7.54)	IV	
Shimazaki Y et al^46^ (2007)	Community-based	584 women	Fasting plasma glucose ≥ 110 (mg/dL)	Mean pocket depth (≥2.0 mm)	Adjusted OR: 2.2 (95% CI: 1.3–3.9)	IV	The Hisayama study
Saito T et al^47^ (2005)	Community-based	584 women	Fasting plasma glucose ≥ 126 or post-challenge ≥ 200 (mg/dL)	Mean pocket depth (≥1.9 mm)	Adjusted OR: 1.4 (95% CI: 0.6–3.2)	IV	The Hisayama study
Saito T et al^48^ (2004)	Community-based	961 subjects	Fasting or 2-h post-challenge plasma glucose levels ≥ 126 mg/dL or 200 mg/dL, respectively	Mean pocket depth (>2.0 mm)	Mean pocket depth (>2.0 mm): 31.7% (32/101) among diabetes, and 17.5% (117/669) among normal glucose tolerance	IV	The Hisayama study

Reference (Publication year)	Design	Study population	Exposure measurement (diabetes)	Outcome measurement	Main results within the scope of the current literature review	Evidence classification^c^	Remarks

**Fracture**							
Tanaka et al^49^ (2013)	Hospital based cohort study	1,614 postmenopausal women	HbA1c ≥ 6.5% or prescribed anti-diabetic drug	Clinically diagnosed	OR for femoral neck fracture: 2.33 (95% CI: 0.96–5.65), OR for vertebral fracture: 0.80 (95% CI: 0.49–1.29), OR for long bone fracture: 1.20 (95% CI: 0.66–2.19)	II	The Nagano Cohort
Tatsuno et al^50^ (2013)	A cross-sectional survey for postmenopausal osteoporosis	64,809 women aged >40 years	Self-reported	Bone mineral density	Adjusted OR for bone fracture: 1.26 (95% CI: 1.05–1.50)	IV	Chiba bone survey
Yamamoto et al^51^ (2007)	Hospital based cross-sectional study	150 female patients with type 2 diabetes and 716 females without diabetes	Clinical diagnosed	Vertebral deformity	OR^b^ for vertebral fracture: 0.74 (95% CI: 0.47–1.17)	IV	Shimane University group
Yamamoto et al^52^ (2009)	Hospital based cross-sectional study	298 patients with type 2 diabetes and 754 patients without diabetes	Clinical diagnosed	Reduction of 20% or more according to the Genant visual criteria	Adjusted OR for vertebral fracture: 1.86 (95% CI: 1.11–3.12) for women and 4.73 (2.19–10.20) for men	IV	Shimane University group
Yamamoto et al^53^ (2012)	Hospital based cross-sectional study	255 patients with type 2 diabetes and 240 patients without diabetes	Clinical diagnosed	Reduction of 20% or more according to the Genant visual criteria	OR^b^ for vertebral fracture: 1.44 (95% CI: 0.85–2.41) for women and 0.69 (95% CI: 0.36–1.31) for men	IV	Shimane University group

Reference (Publication year)	Design	Study population	Exposure measurement (diabetes)	Outcome measurement	Main results within the scope of the current literature review	Evidence classification^c^	Remarks

**Impaired cognitive function**							
Ohara et al^54^ (2011)	Cohort study for 15 years	1017 community-dwelling dementia-free subjects	Fasting plasma glucose ≥ 7.0 mmol/L or 2-h plasma glucose level ≥ 11.1 mmol/L	All-cause dementia	Adjusted HR: 1.74 (95% CI: 1.19–2.53) for all-cause dementia, 2.05 (95% CI: 1.18–3.57) for Altzheimer disease, and 1.82 (95% CI: 0.89–3.71) for vascular dementia	II	The Hisayama study
Doi et al^55^ (2015)	Community-based	9683 older adults	Interviewed about the presence or absence of diabetes	Motoric cognitive risk syndrome (MCR)	Adjusted OR for motoric cognitive risk syndrome: 1.47 (95% CI: 1.18–1.85)	IV	National Center for Geriatrics and Gerontology Study of Geriatric Syndromes
Shimada et al^56^ (2010)	Hospital based cross-sectional study	103 diabetes patients	Hospitalized diabetes patients	Amnestic mild cognitive impairment	ORs: 7.87 (95% CI: 1.28–47.55) for dementia,1.41 (95% CI: 0.56–3.55) for dementia and amnestic MCI	IV	
Mogi et al^57^ (2004)	Hospital based cross-sectional study	69 diabetes patients	Diabetes outpatients	Mini-Mental State Examination	MMSE: 27.1 among diabetes patients and 28.3 among non-diabetes patients (*P* < 0.05)	IV	

Reference (Publication year)	Design	Study population	Exposure measurement (diabetes)	Outcome measurement	Main results within the scope of the current literature review	Evidence classification^c^	Remarks

**Depression**							
Takasaki et al^58^ (2008)	Cross-sectional study for seven community population	832 subjects	Self-reported	World Health Organization Composite International Diagnostic Interview (WMH-CIDI 3.0)	Crude OR: 1.57 (95%CI: 0.59–4.21), adjusted OR: 2.15 (95% CI: 0.62–7.49)	III	
Arima et al^59^ (2008)	Cross-sectional study	6543 subjects (195 had type 2 diabetes)	Medical claim (ICD10 code, E11)	Medical claim (ICD10 code, F32, F33, and F34)	Crude OR: 2.20 (95%CI: 0.80–5.50), adjusted OR: 2.33 (95% CI: 0.86–6.33)	IV	

HR, hazard ratio; OR, Odds ratio; IRR, incidence rate ratio; RR, risk ratio.

^a^We extracted data that were within the scope of the current literature review.

^b^Odds ratios were calculated from the data in the cross table by the authors of this literature review.

^c^I: Integrated analyses, II: Cohort study design, III: Case-control study design (longitudinal), IV: Cross-sectional study design.

### Periodontal disease and diabetes

Eleven studies on the subject of periodontal disease in diabetes were identified through title and abstract review (nine from PubMed and two from additional searches) and were reviewed in detail. After evaluating entire texts, we found one cohort study and four cross-sectional studies, including one study conducted as a case-control study but in which diabetes and periodontal disease status were measured at the same time. We found no integrated analyses (Table 2).

Morita et al reported a cohort study of 5,856 participants (workers in and around Nagoya City) who were followed up for 5 years, focusing on 150 patients (2.6%) with HbA1c ≥6.5% at baseline.^44^ The RR for developing periodontal pockets (a Community Periodontal Index of 3 or 4) was 1.17 (95% CI, 1.01–1.36) in those with HbA1c ≥6.5% at baseline, after adjustment for body mass index, smoking status, sex, and age.^44^ In another study, Marugame et al evaluated alveolar bone loss and showed an adjusted OR of 2.55 (95% CI, 0.86–7.54) for the complication among 664 Japanese men aged 46–57 years with newly diagnosed type 2 diabetes.^45^

Three studies^46^^–^^48^ used secondary data analyses from the same prospective cohort (the Hisayama study). The Hisayama study comprised a series of prospective studies of cerebrovascular-cardiovascular disease initiated in 1961 in Hisayama, a suburban town in the Fukuoka metropolitan area of Kyushu Island in Japan. Two studies indicated that there were significant associations between the prevalence of periodontal diseases and impaired fasting glucose (IFG),^46^ impaired glucose tolerance (IGT), and diabetes in women.^47^ Moreover, Saito et al showed that periodontal disease is associated with the development of glucose intolerance.^48^ Despite using data from prospective cohort studies, the estimates derived for the present study were obtained from cross-sectional investigations (Table 3).^46^^–^^48^

### Fractures and diabetes

We reviewed 21 titles and abstracts concerning the relationship between diabetes and fractures. This revealed one cohort study, four cross-sectional studies, and no integrated analyses (Table 2).

In a hospital-based cohort study from the Nagano Cohort, the authors reported the follow-up data for 1,614 postmenopausal women (8.1% had diabetes) over a median period of 6.7 years to evaluate the association between lifestyle-related diseases and fractures. The ORs for fracture among patients with diabetes compared with those without diabetes were 2.33 (95% CI, 0.96–5.65) for femoral neck fracture, 0.80 (95% CI, 0.49–1.29) for vertebral fracture, and 1.20 (95% CI, 0.66–2.19) for long bone fracture.^49^

A population-based, multicenter, cross-sectional survey investigating postmenopausal osteoporosis in Chiba City was reported for the period from 2001 to 2009 (the Chiba Bone Survey).^50^ Among the 64,809 women aged >40 years, the authors identified that 2,116 had diabetes and that osteoporosis (BMD <74% of young adult mean) was present in 22.5% of patients with diabetes and 15.6% of individuals without diabetes. Fracture during the most recent 5 years occurred in 7.6% of patients with diabetes, which was significantly higher than the rate of 5.2% that occurred in patients without diabetes (*P* < 0.001). The adjusted OR for bone fracture during the most recent 5 years was 1.26 (95% CI, 1.05–1.50) in patients with diabetes.

There were also three hospital-based studies conducted by the Shimane University group, but it should be noted that there may have been an overlap of the study participants.^51^^–^^53^ In one of these studies, 298 patients with type 2 diabetes were compared with nondiabetic controls.^52^ Among patients with type 2 diabetes, 43 women (31.4%) and 61 men (37.9%) had vertebral fractures, whereas in the control group, the percentages were 24.9% and 14.5%, respectively. Logistic regression analysis showed that type 2 diabetes was an independent risk factor for the prevalence of vertebral fracture in women (OR 1.86; 95% CI, 1.11–3.12) and men (OR 4.73; 95% CI, 2.19–10.20) after adjustment for age, body mass index, and BMD of the lumber spine (Table 3).^52^

### Impaired cognitive function and diabetes

We reviewed 14 titles and abstracts reporting the association between diabetes and impaired cognitive function, including mild cognitive impairment (MCI) in Japanese populations. This revealed one cohort study and three cross-sectional studies (Table 2).

Population-based evidence was reported from the Hisayama study.^54^ In the study, community-dwelling subjects with dementia who were aged 60 years or older (*n* = 1,017) were divided into five groups according to the results of a 75-g oral glucose tolerance test (normal glucose tolerance, IFG, IGT, both IFG and IGT, and diabetes).^54^ Over a 15-year follow-up period, diabetes was shown to be a significant risk factor for the development of all-cause dementia (adjusted HR 1.74; 95% CI, 1.19–2.53), Alzheimer’s disease (adjusted HR 2.05; 95% CI, 1.18–3.57), and vascular dementia (adjusted HR 1.82; 95% CI, 0.89–3.71).

A cross-sectional study was carried out by Doi et al to reveal the prevalence and risk factors of motoric cognitive risk (MCR) syndrome. They aimed to report the prevalence of, and modifiable factors associated with, MCR in Japanese community-dwelling adults and screened 9,683 older adults (52% women, mean age: 73.6 years). Among the patients with MCR, 20.4% had diabetes, and among the patients without MCR, 12.4% had diabetes (*P* < 0.001). After adjusting for several covariates, diabetes was associated with an increased risk of MCR (OR 1.47; 95% CI, 1.18–1.85).^55^

The prevalence of MCI was investigated in another cross-sectional study. Among 103 patients with diabetes, 22 (21%) had dementia and 9 (9%) had amnestic MCI, while in a group of 30 controls, 1 (3%) had dementia and 6 (20%) had amnestic MCI. The ORs were 7.87 (95% CI, 1.28–47.55) for dementia and 1.41 (95% CI, 0.56–3.55) for dementia and amnestic MCI.^56^ The final cross-sectional study investigated cognitive function in Japanese patients with diabetes (*n* = 69) and showed that they had significantly worse Mini-Mental State Examination scores than those without diabetes (*n* = 27) (27.1 and 28.3, respectively; *P* < 0.05) (Table 3).^57^

### Depression and diabetes

We reviewed 18 titles and abstracts for studies reporting the association between diabetes and depression. Among these, there was one case-control study and one cross-sectional study that met our inclusion criteria (Table 2).

Takasaki et al reported a community-based case-control study (World Mental Health Japan, 2002–2004) of two urban and five rural community areas in which they examined the association between depression and circulatory diseases, including diabetes.^58^ They used the World Health Organization Composite International Diagnostic Interview Version 3.0 to assess major depression. The reported crude OR was 1.57 (95% CI, 0.59–4.21), with diabetes being associated with 2.15-fold higher odds of developing major depression after adjusting for sex, age, smoking status, alcohol intake, and education; however, the association was not statistically significant (95% CI, 0.62–7.49). In the cross-sectional study, claims data and a self-reported questionnaire were used for 6,543 Japanese employees aged 18–65 years (195 with type 2 diabetes and 6,348 without type 2 diabetes).^59^ The prevalence of comorbid depression was 2.6% in the group with diabetes but only 1.2% in the group without diabetes (crude OR 2.20; 95% CI, 0.88–5.50). After adjustment for age, gender, alcohol intake, smoking status, exercise, and dietary restriction, the OR for depression in type 2 diabetes was 2.33 (95% CI, 0.86–6.33) (Table 3).

## DISCUSSION

### Main findings

We reviewed studies in Japan that examined comorbid conditions recently linked to diabetes. Although there were several cohort studies and meta-analyses regarding the relationship for cancer in diabetes, there was scant epidemiological evidence for the other four conditions. Indeed, only one cohort study each was identified for periodontal disease, fracture, and cognitive impairment. The remaining evidence was either cross-sectional or collected from tables of baseline characteristics in studies with other aims. However, some cohort studies had notable limitations, particularly in terms of the study setting and sample size. Regarding the evaluation of the Newcastle-Ottawa scale, the scores were high in both cohort and case-control studies; however, we found it difficult to judge the overall tendency due to the inadequate number of studies. Overall, our results underscore the need for more epidemiological evidence about these newly recognized comorbidities in Japan. Only then can we gain valid insights to help us plan effective management of these conditions. By conducting this systematic review of epidemiological studies, we have highlighted the areas where the best research has been done, thereby highlighting which conditions future epidemiological studies should target. We believe that further epidemiological studies will help to obtain detailed information on the influence of diabetes on comorbidities, which would contribute to forthcoming revision of guidelines for diabetes care in Japan.

### Interpretation

For cancer, we found epidemiological studies of relatively high quality investigating the relationship with diabetes. Several studies, such as the JPHC study, were community-based and well designed and had good generalizability to the general Japanese population. However, some research questions remain unresolved. For example, diabetes in general has been reported to be protective for prostate cancer in many non-Japanese populations,^60^ but we found the opposite effect in the Japanese population.^37^ The reason for this discrepancy may be that the impact of diabetes on cancer is different in Asian and western populations. Further research in Japanese populations would help clarify this relationship, and there are several ongoing prospective cohort studies in Japan.^61^^–^^63^ Further observation and analysis should provide clarifying epidemiological evidence.

In contrast to the range of studies for cancer, there were quite limited numbers of studies for the other four conditions. The Hisayama study, a population-based prospective cohort study, was notable among these, demonstrating that a history of diabetes was a significant risk factor for the development of all-cause dementia, Alzheimer’s disease, and vascular dementia. A part of the Hisayama study was included in a pooled international analysis that showed that diabetes patients have approximately 60–70% greater risk for the development of dementia compared with those without diabetes.^19^ However, there was a paucity of good-quality cohort studies for periodontal disease and fracture, with some being old and/or having small sample sizes and others being hospital-based and lacking generalizability. For example, although Morita et al tried to investigate the relationship between diabetes and periodontal disease, their results should be interpreted with caution in terms of external validity because the cohort was derived from employed workers receiving annual medical checkups. By contrast, a population-based cohort study of 2,626 community-dwelling Germans in 2012 substantially advanced previous research on the influence of etiology and glycemic control on periodontal disease, using population-based longitudinal data.^64^ Therefore, we concluded that the studies investigating the relationship between diabetes and periodontal disease, fracture, and depression in Japan have been too few to compare with studies in other countries. Well-designed prospective cohort studies can help understand the newly recognized diabetes-related conditions among patients in Japan.

Some efforts could increase the quality and quantity of epidemiological evidence in this field. First, when researchers investigate the relationship between diabetes as an exposure and the complications as the outcome in cohort studies, they need to follow the occurrence of these complications in participants. To make it easy to capture the occurrence, the development of valid and feasible measurements is necessary. For example, to follow the occurrence of periodontal diseases, some studies have used the Community Periodontal Index,^41^ whereas others have used alveolar bone loss.^45^ Like these criteria, it is important to give explicit criteria for the diagnosis of complications if we are to ensure optimal intra- and inter-rater reliability that will improve the interpretability of results. Moreover, establishing common criteria will facilitate the implementation of epidemiological studies by rendering study design easier and between-study comparison more straightforward.

Second, utilizing existing cohorts will lower the cost and time needed to obtain new evidence. Some complications may also be too expensive to follow up for all the cohort members; in this case, researchers may want to use more efficient study designs, such as a case-cohort design.

Third, utilization of existing data (eg, claims data) may be another efficient way to obtain clinical data, although it has inherent limitations. Antidiabetic medicine use can be utilized to detect individuals with diabetes with high specificity; however, it can misclassify patients not using medication. In addition, detecting periodontal disease and dementia may be almost impossible.

Given the limitations of current epidemiological studies, combining insights gained from several types of studies is necessary to grasp the full landscape of diabetes and its complications. Furthermore, the strategic allocation of research grants in this field is needed to conduct more studies of higher quality.

### Limitations

There are several limitations in this study. First, although we attempted to include all studies of interest by conducting an extensive systematic review, we may have failed to include a few studies, especially those with cross-sectional designs. This is because some of our cross-sectional evidence came from the tables of baseline characteristics in studies done for other reasons. Second, we did not include evidence from single-arm studies, such as those that estimated the incidence of comorbid conditions among patients with diabetes only. We decided not to include these studies because they may lack comparability with other studies. If comparability could be ensured, combining single-arm studies could be an efficient strategy for estimating incidence rates. For example, the Food and Drug Administration allows registry data to be used to provide historical controls to replace the post-approval study requirement for pharmaceutical approval reviews.^65^ Combining a single-arm clinical trial with historical controls from existing databases during the pharmaceutical application process is analogous to combining a single-arm epidemiological cohort of patients with diabetes and a community-based cohort that is not restricted to patients with diabetes. This could generate epidemiological findings about the incidence of comorbid conditions among patients with diabetes compared with the general population. Third, evidence of inverse causal relationships (ie, from comorbid conditions to diabetes) was beyond the scope of this study. Because it is highly likely that the five comorbidities we dealt with could lead to the development of diabetes or the worsening of glycemic control, further review might be needed to illuminate the whole relationship between diabetes and its comorbidities.

### Conclusion

In Japan, there is scant evidence about the effect of diabetes on the incidence and prevalence of periodontal disease, fracture, impaired cognitive function and dementia, and depression. By contrast, there is much stronger evidence for the relationship of diabetes with cancer. Future efforts are needed to improve the quality and quantity of evidence specific to the Japanese population if we are to provide evidence-based interventions against these putative complications of diabetes.

## APPENDIX A. SUPPLEMENTARY DATA

eMaterial 1. PRISMA 2009 Checklist

eTable 1. The search terms used for PubMed with the MeSH terms

eTable 2. Newcastle-Otawa Scale (cohort studies)

eTable 3. Newcastle-Otawa Scale (case-control studies)

eFigure 1. PRISMA flow diagram and number of records identified for diabetes and cancer

eFigure 2. PRISMA flow diagram and number of records identified for diabetes and periodontal disease

eFigure 3. PRISMA flow diagram and number of records identified for diabetes and fracture

eFigure 4. PRISMA flow diagram and number of records identified for diabetes and dementia

eFigure 5. PRISMA flow diagram and number of records identified for diabetes and depression
