# Supplementary material for: Contribution of Diabetes to the Incidence and Prevalence of Comorbid Conditions (Cancer, Periodontal Disease, Fracture, Impaired Cognitive Function, and Depression): A Systematic Review of Epidemiological Studies in Japanese Populations
Source: J Epidemiol. 2019 Jan 5;29(1):1–10. doi: 10.2188/jea.JE20170155 (PMC6290274; doi:10.2188/jea.JE20170155)
Supplement: Supplementary file 1 [file je-29-001-s001.pdf]

eMaterial 1. PRISMA 2009 Checklist

Section/topic	#	Checklist item	Reported on page #
**TITLE**			
Title	1	Identify the report as a systematic review, meta-analysis, or both.	1
**ABSTRACT**			
Structured summary	2	Provide a structured summary including, as applicable: background; objectives; data sources; study eligibility criteria, participants, and interventions; study appraisal and synthesis methods; results; limitations; conclusions and implications of key findings; systematic review registration number.	3-4
**INTRODUCTION**			
Rationale	3	Describe the rationale for the review in the context of what is already known.	5-11
Objectives	4	Provide an explicit statement of questions being addressed with reference to participants, interventions, comparisons, outcomes, and study design (PICOS).	5-11
**METHODS**			
Protocol and registration	5	Indicate if a review protocol exists, if and where it can be accessed (e.g., Web address), and, if available, provide registration information including registration number.	None
Eligibility criteria	6	Specify study characteristics (e.g., PICOS, length of follow-up) and report characteristics (e.g., years considered, language, publication status) used as criteria for eligibility, giving rationale.	11-12
Information sources	7	Describe all information sources (e.g., databases with dates of coverage, contact with study authors to identify additional studies) in the search and date last searched.	11-12
Search	8	Present full electronic search strategy for at least one database, including any limits used, such that it could be repeated.	11-12
Study selection	9	State the process for selecting studies (i.e., screening, eligibility, included in systematic review, and, if applicable, included in the meta-analysis).	12-14
Data collection process	10	Describe method of data extraction from reports (e.g., piloted forms, independently, in duplicate) and any processes for obtaining and confirming data from investigators.	12-14
Data items	11	List and define all variables for which data were sought (e.g., PICOS, funding sources) and any assumptions and simplifications made.	12-14
Risk of bias in individual studies	12	Describe methods used for assessing risk of bias of individual studies (including specification of whether this was done at the study or outcome level), and how this information is to be used in any data synthesis.	12-14
Summary measures	13	State the principal summary measures (e.g., risk ratio, difference in means).	12-13
Synthesis of results	14	Describe the methods of handling data and combining results of studies, if done, including measures of consistency (e.g., I^2) for each meta-analysis.	None

eMaterial 1. PRISMA 2009 Checklist

Section/topic	#	Checklist item	Reported on page #
Risk of bias across studies	15	Specify any assessment of risk of bias that may affect the cumulative evidence (e.g., publication bias, selective reporting within studies).	None
Additional analyses	16	Describe methods of additional analyses (e.g., sensitivity or subgroup analyses, meta-regression), if done, indicating which were pre-specified.	None
**RESULTS**			
Study selection	17	Give numbers of studies screened, assessed for eligibility, and included in the review, with reasons for exclusions at each stage, ideally with a flow diagram.	14-21
Study characteristics	18	For each study, present characteristics for which data were extracted (e.g., study size, PICOS, follow-up period) and provide the citations.	14-21
Risk of bias within studies	19	Present data on risk of bias of each study and, if available, any outcome level assessment (see item 12).	14-21
Results of individual studies	20	For all outcomes considered (benefits or harms), present, for each study: (a) simple summary data for each intervention group (b) effect estimates and confidence intervals, ideally with a forest plot.	14-21
Synthesis of results	21	Present results of each meta-analysis done, including confidence intervals and measures of consistency.	None
Risk of bias across studies	22	Present results of any assessment of risk of bias across studies (see Item 15).	None
Additional analysis	23	Give results of additional analyses, if done (e.g., sensitivity or subgroup analyses, meta-regression [see Item 16]).	None
**DISCUSSION**			
Summary of evidence	24	Summarize the main findings including the strength of evidence for each main outcome; consider their relevance to key groups (e.g., healthcare providers, users, and policy makers).	21-22
Limitations	25	Discuss limitations at study and outcome level (e.g., risk of bias), and at review-level (e.g., incomplete retrieval of identified research, reporting bias).	25-26
Conclusions	26	Provide a general interpretation of the results in the context of other evidence, and implications for future research.	27
**FUNDING**			
Funding	27	Describe sources of funding for the systematic review and other support (e.g., supply of data); role of funders for the systematic review.	27

From: Moher D, Liberati A, Tetzlaff J, Altman DG, The PRISMA Group (2009). Preferred Reporting Items for Systematic Reviews and Meta-Analyses: The PRISMA Statement. PLoS Med 6(7): e1000097. doi:10.1371/journal.pmed1000097

For more information, visit: **www.prisma-statement.org**.

eTable 1. The search terms used for PubMed with the MeSH terms

Common comorbid conditions	Search results*	Reviewed literature in detail	Identified literature
**Cancer**			
(neoplasms[MeSH] or cancer)<br>(Diabetes Mellitus[MeSH] or diabetes)<br>(Japan* or Japan[MeSH])<br>(prevalence[MeSH] or prevalence or incidence[MeSH] or incidence)	770	67	17
**Periodontal disease**			
(periodontal diseases[MeSH] or periodontal)<br>(Diabetes Mellitus[MeSH] or diabetes)<br>(Japan* or Japan[MeSH])	181	11	5
**Fracture**			
(fractures[MeSH] or fractures or osteoporosis[MeSH] or osteoporosis)<br>(Diabetes Mellitus[MeSH] or diabetes)<br>(Japan* or Japan[MeSH])<br>(prevalence[MeSH] or prevalence or incidence[MeSH] or incidence)	88	21	5
**Dementia**			
(dementia[MeSH] or dementia or cognitive)<br>(Diabetes Mellitus[MeSH] or diabetes)<br>(Japan* or Japan[MeSH])<br>(prevalence[MeSH] or prevalence or incidence[MeSH] or incidence)	186	14	4
**Depression**			
(depression[MeSH] or depression)<br>(Diabetes Mellitus[MeSH] or diabetes)<br>(Japan* or Japan[MeSH])<br>(prevalence[MeSH] or prevalence or incidence[MeSH] or incidence)	116	18	2

eTable 2. Newcastle-Otawa Scale (cohort studies)

Reference (Publication year)	Selection				Comparability		Outcome			Remarks
	1) Representativeness of the exposed cohort	2) Selection of the non exposed cohort	3) Ascertainment of exposure	4) Demonstration that outcome of interest was not present at start of study	1) Comparability of cohorts on the basis of the design or analysis: Controls for Age or Sex	1) Comparability of cohorts on the basis of the design or analysis: Controls for Boby Mass Index	1) Assessment of outcome	2) Was follow-up long enough for outcomes to occur	3) Adequacy of follow up of cohorts	
**Cancer**										
Inoue et al.[30] (2006)	*	*		*	* (by sex)	*	*	*	*	The JPHC study
Goto et al.[31] (2016)	*	*	*	*	*	*	*	*	*	
Nakamura et al.[32] (2013)	*	*		*	* (by sex)	*	*	*	*	The Takayama study
Khan et al.[33] (2006)	*	*		*	* (by sex)	*	*	*	*	The JACC study
Luo et al.[34] (2007)	*	*		*	* (by sex)	*	*	*	*	The JPHC study
Ikeda et al.[35] (2009)	*	*	*	*	*	*	*	*	*	The Hisayama study
Sekikawa et al.[36] (2014)		*	*	*	*		*	*	*	
Li et al.[37] (2010)	*	*		*	* (men only)	*	*	*	*	The Ohsaki Cohort Study
**Periodontal disease**										
Morita I et al.[44] (2012)	*	*	*	*	*	*	*	*	*	
**Fracture**										
Tanaka et al.[49] (2013)	*	*	*	*	* (postmenopausal women, but not age adusted）		*	*	*	The Nagano Cohort
**Impaired cognitive function**										
Ohara et al.[54] (2011)	*	*	*	*	*	*	*	*	*	The Hisayama study

eTable 3. Newcastle-Otawa Scale (case-control studies)

Reference (Publication year)	Selection				Comparability		Exposure			Remarks
	1) Is the case definition adequate?	2) Representativeness of the cases	3) Selection of Controls	4) Definition of Controls	1) Comparability of cohorts on the Controls for Age or Sex	Controls for Boby Mass Index	1) Ascertainment of exposure	2) Same method of ascertainment for cases and	3) Non-Response rate	
**Cancer**										
Kuriki et al.[38] (2007)		*		*	*	*	*	*		The Hospital-based Epidemiologic Research Program at Aichi Cancer Center
Ohishi et al.[39] (2008)	*	*	*	*	*	*	*	*	*	The Adult Health Study longitudinal cohort (cohort of atomic bomb survivors)
Matsuo et al.[40] (2003)		*		*	* (by sex)			*		
Inoue et al.[41] (2003)		*		*	* (by sex)			*	*	The Hospital-based Epidemiologic Research Program at Aichi Cancer Center
Mori et al.[42] (1998)	*	*	*	*	* (women only)		*	*		
**Depression**										
Takasaki et al.[58] (2008)	*	*	*	*	*		*	a	b	

PRISMA 2009 Flow Diagram

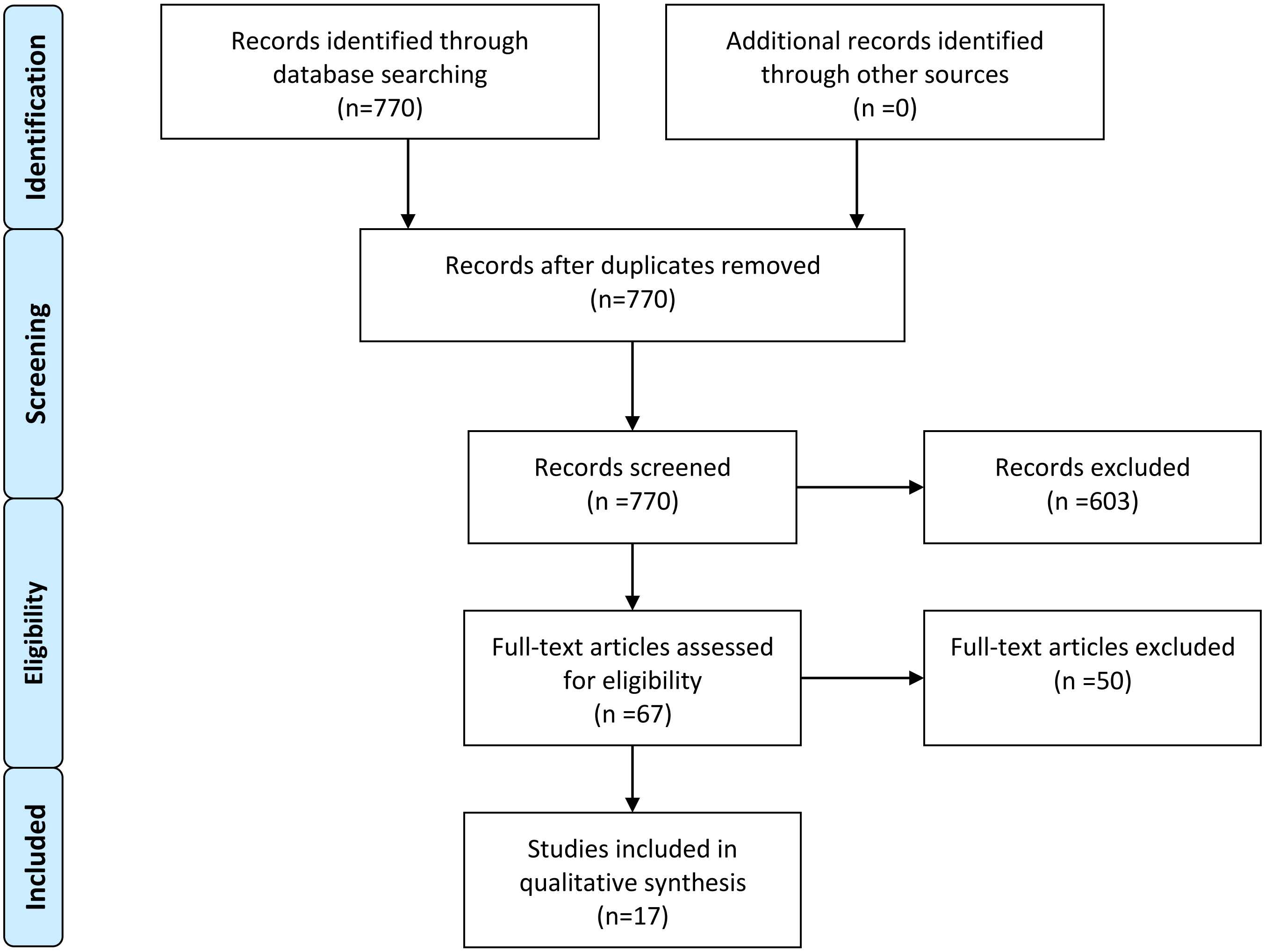

eFigure 1. PRISMA flow diagram and number of records identified for diabetes and cancer

PRISMA 2009 Flow Diagram

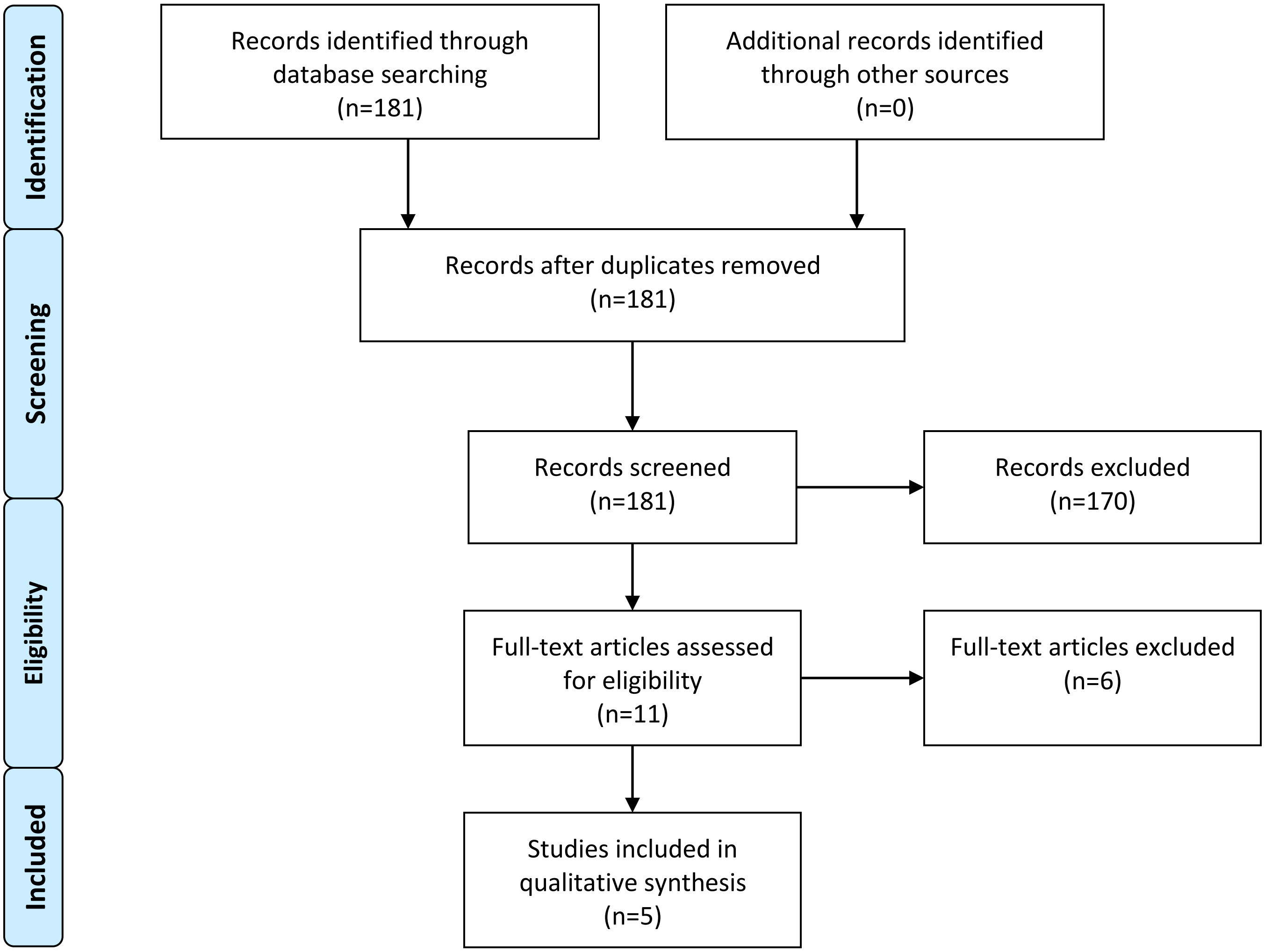

eFigure 2. PRISMA flow diagram and number of records identified for diabetes and periodontal disease

From: Moher D, Liberati A, Tetzlaff J, Altman DG, The PRISMA Group (2009). *P*referred *R*eporting *I*tems for *S*ystematic Reviews and *M*eta-*A*nalyses: The PRISMA Statement. PLoS Med 6(7): e1000097. doi:10.1371/journal.pmed1000097

For more information, visit www.prisma-statement.org.

PRISMA 2009 Flow Diagram

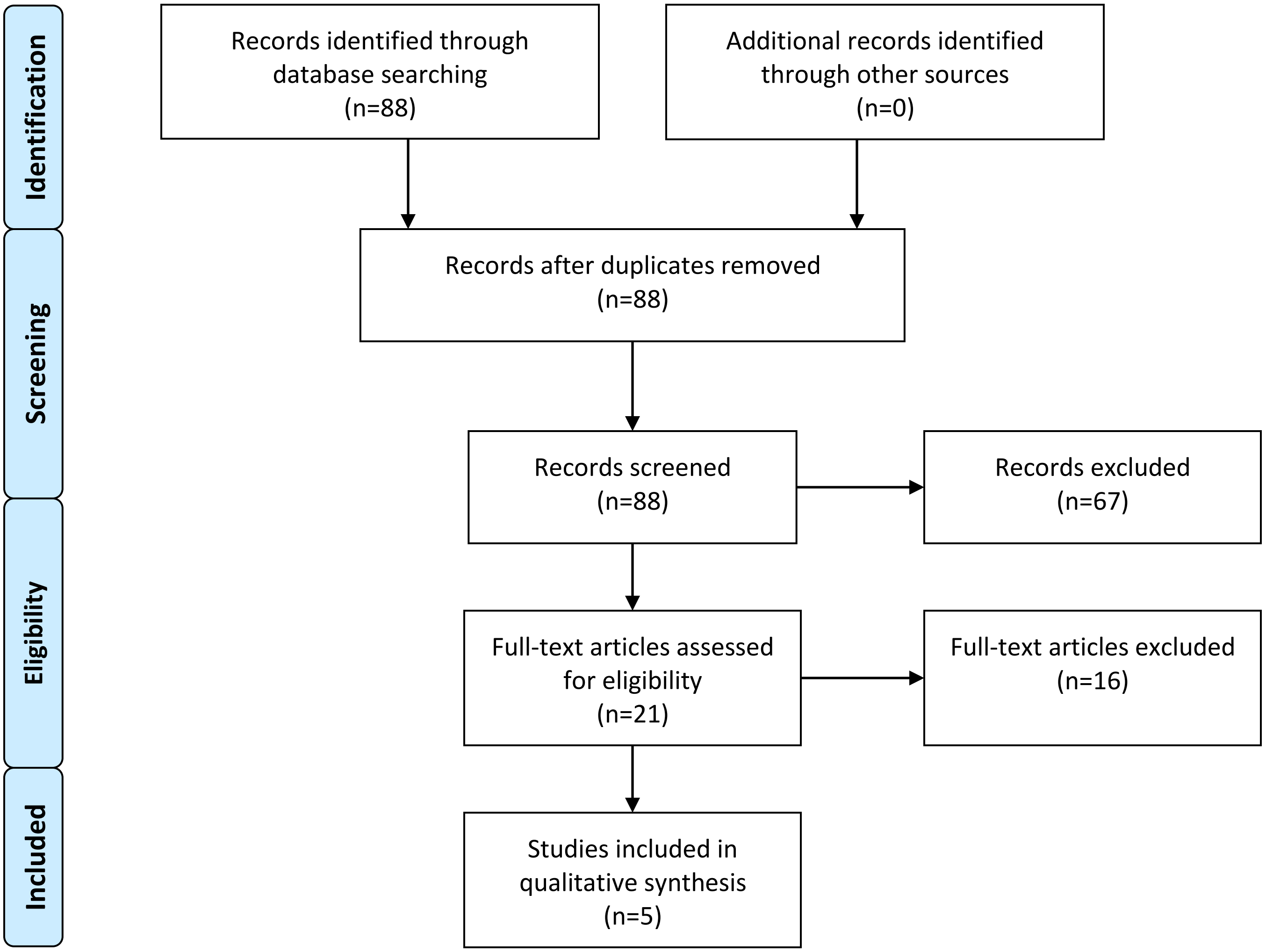

eFigure 3. PRISMA flow diagram and number of records identified for diabetes and fracture

From: Moher D, Liberati A, Tetzlaff J, Altman DG, The PRISMA Group (2009). *P*referred *R*eporting *I*tems for *S*ystematic Reviews and *M*eta-*A*nalyses: The PRISMA Statement. PLoS Med 6(7): e1000097. doi:10.1371/journal.pmed1000097

PRISMA 2009 Flow Diagram

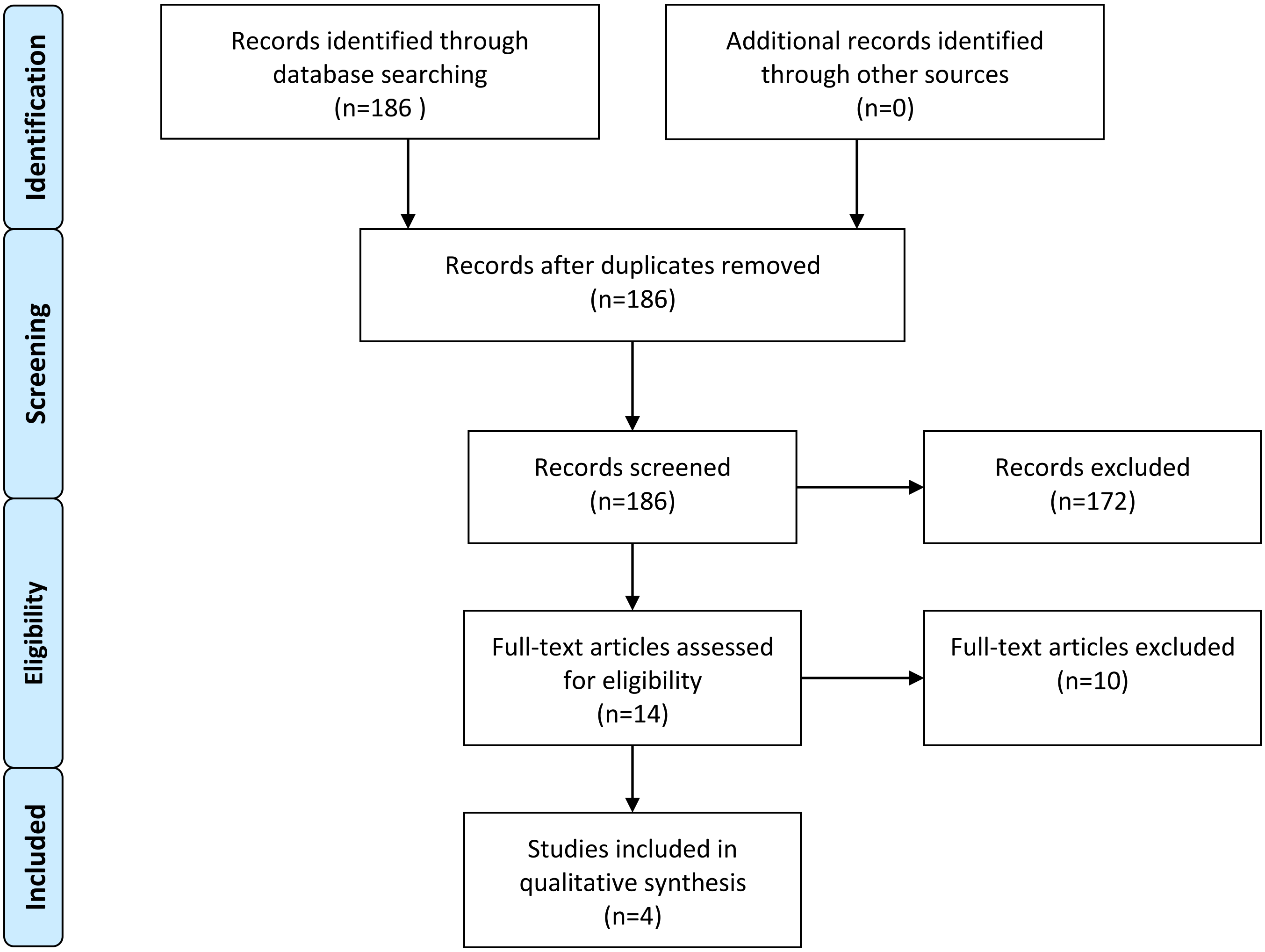

eFigure 4. PRISMA flow diagram and number of records identified for diabetes and dementia

From: Moher D, Liberati A, Tetzlaff J, Altman DG, The PRISMA Group (2009). *P*referred *R*eporting *I*tems for *S*ystematic Reviews and *M*eta-*A*nalyses: The PRISMA Statement. PLoS Med 6(7): e1000097. doi:10.1371/journal.pmed1000097

For more information, visit www.prisma-statement.org.

PRISMA 2009 Flow Diagram

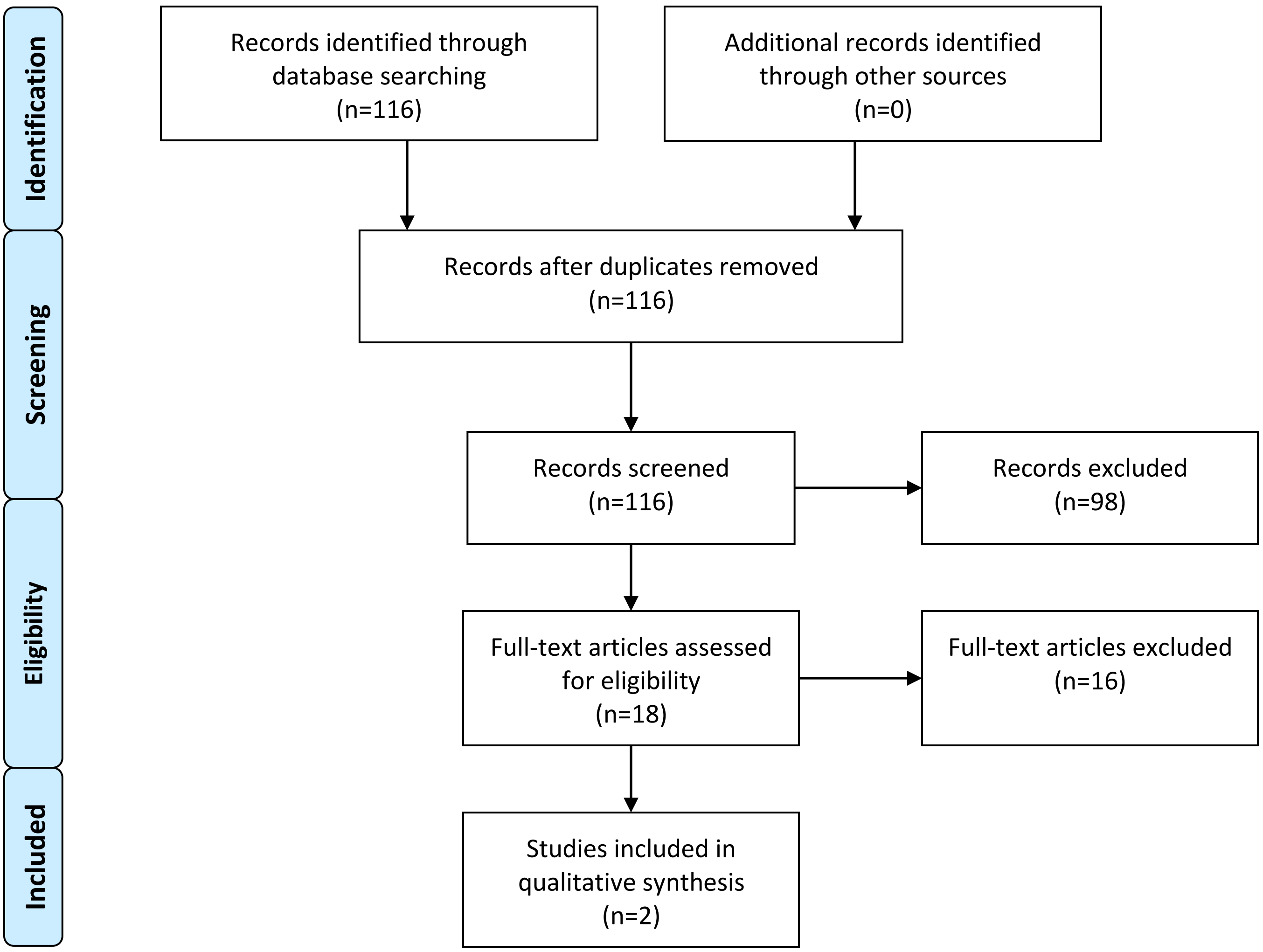

eFigure 5. PRISMA flow diagram and number of records identified for diabetes and depression

From: Moher D, Liberati A, Tetzlaff J, Altman DG, The PRISMA Group (2009). *P*referred *R*eporting *I*tems for *S*ystematic Reviews and *M*eta-*A*nalyses: The PRISMA Statement. PLoS Med 6(7): e1000097. doi:10.1371/journal.pmed1000097

For more information, visit www.prisma-statement.org.
